# The wandering mood: psychological and neural determinants of rest-related negative affect

**DOI:** 10.3389/fpsyg.2013.00961

**Published:** 2013-12-26

**Authors:** Michal Gruberger, Adi Maron-Katz, Haggai Sharon, Talma Hendler, Eti Ben-Simon

**Affiliations:** ^1^Functional Brain Center, Wohl Institute for Advanced Imaging, Tel Aviv Sourasky Medical Center, Tel Aviv UniversityTel Aviv, Israel; ^2^School of Psychological Sciences, Tel Aviv UniversityTel Aviv, Israel; ^3^Sackler Faculty of Medicine, Tel Aviv UniversityTel Aviv, Israel

**Keywords:** affect, anterior cingulate cortex, default mode network, executive network, mind wandering, resting state functional connectivity, salience network, vigilance

## Abstract

Rest related negative affect (RRNA) has gained scientific interest in the past decade. However, it is mostly studied within the context of mind-wandering (MW), and the relevance of other psychological and neural aspects of the resting state to its’ occurrence has never been studied. Several indications associate RRNA with internally directed attention, yet the nature of this relation remains largely unknown. Moreover, the role of neural networks associated with rest related phenomenology – the default mode (DMN), executive (EXE), and salience (SAL) networks, has not been studied in this context. To this end, we explored two 5 (baseline) and 15-minute resting-state simultaneous fMRI-EEG scans of 29 participants. As vigilance has been shown to affect attention, and thus its availability for inward allocation, EEG-based vigilance levels were computed for each participant. Questionnaires for affective assessment were administered before and after scans, and retrospective reports of MW were additionally collected. Results revealed increased negative affect following rest, but only among participants who retained high vigilance levels. Among low-vigilance participants, changes in negative affect were negligible, despite reports of MW occurrence in both groups. In addition, in the high-vigilance group only, a significant increase in functional connectivity (FC) levels was found between the DMN-related ventral anterior cingulate cortex (ACC), associated with emotional processing, and the EXE-related dorsal ACC, associated with monitoring of self and other’s behavior. These heightened FC levels further correlated with reported negative affect among this group. Taken together, these results demonstrate that, rather than an unavoidable outcome of the resting state, RRNA depends on internal allocation of attention at rest. Results are discussed in terms of two rest-related possible scenarios which defer in mental and neural processing, and subsequently, in the occurrence of RRNA.

## INTRODUCTION

Recent years in cognitive neuroscience have seen a major paradigm shift from emphasizing task related neural activity to exploring rest related neural and mental states. Within a relatively short period, a substantial body of findings led to the recognition that the study of the resting state is necessary for a more complete understanding of human psychological functioning and its underlying neural mechanisms. Within rest related cognition, most efforts have focused on studying mind-wandering (MW), which may be defined as ongoing mentation that occurs spontaneously, and largely autonomously, whenever an awake individual is either at rest or not fully concentrated during performance of a task (Smallwood and Schooler, 2006; Baars, 2010; Gruberger et al., 2011; Andrews-Hanna, 2012; Poerio et al., 2013). Importantly, “rest” in this context does not imply a complete lack of a task (Giambra, 1995), but rather a state in which attention load is relatively small. Efforts to systematically study MW have indeed demonstrated that humans tend to mind-wander at least half of their waking hours (Klinger and Cox, 1987; Killingsworth and Gilbert, 2010), and that the occurrence of MW is inversely related to the degree of cognitive processing (Smallwood and Schooler, 2006).

Though most studies of rest-related mental functioning have focused on MW, a related psychological phenomenon that has been recurrently documented is rest related negative affect (RRNA). For instance, Killingsworth and Gilbert (2010) sampled a large number of participants during their everyday routines, and demonstrated that not only do people spend as much time thinking about what is not happening as they do about what is happening, but also that when not mentally occupied by the activity taking place they report being less happy. McVay et al. (2009) demonstrated that while individuals are “off-task,” they report worse performance, and that among individuals with a high tendency to MW, being engaged in “off-task” thinking is associated with less happiness. Furthermore, Marchetti et al. (2012) found that reports of being “off-task” predicted subsequent, but not simultaneous, accessibility of negative thoughts, though this finding was confined to individuals with higher levels of depressive symptoms. Based on these findings, the authors suggest that MW may only be “indirectly” associated with negative thoughts. Ottaviani and Couyoumdjian (2013) found that the reported frequency of MW during a low-demand task was even correlated with subsequent increased heart-rate and difficulty falling asleep, though these effects were found to be short-term.

To date, RRNA has mostly been studied in the context of MW. The exact relation between RRNA and MW is, however, a matter of debate. For instance, Killingsworth and Gilbert (2010) present findings that suggest that MW causes negative affect, whereas Stawarczyk et al. (2013) and Smallwood et al. (2009), as well as Poerio et al. (2013), present results advocating for an inverse direction of causation, in which higher negative affect precedes the incidence of MW. Moreover, the degree of immediacy of the relation between these two factors has been called into question, by demonstrating that MW was associated with subsequent accessibility of negative thoughts, but not with concurrent negative thinking (Marchetti et al., 2012). In another study, Michael et al. (2013) demonstrated that it is trait levels of MW that are actually associated with decreased moods among young individuals. Factors relating to the content of MW, such as it’s affective content (Poerio et al., 2013) as well as it’s temporal and social orientation (Ruby et al., 2013), have further been proposed as mediating the association between MW and possible subsequent negative mood. Thus, there seems to be a consensus that when cognitive load allows, MW and negative affect are found to occur (in varying degrees), influence each other and perhaps interact to create further effects. Nevertheless, the ambiguity regarding the relation between these two rest-related phenomena suggests that rest-related negative affect might be a phenomenon of its own, and not merely a simple bi-product of MW. Thus far, to the best of our knowledge, no research has selectively studied the circumstances under which rest-related negative affect arises. Moreover, knowledge of the neural processes that mediate its occurrence is scarce.

Though direct empirical evidence is scarce, several indications exist for a potential role of the direction of attention inward in the ultimate occurrence of RRNA. For instance, Killingsworth and Gilbert (2010) found that overall, mood was better predicted by what people were thinking than by what people were doing. Moreover, even when performing the least enjoyable activities, people reported being less happy if they were mind wandering compared to if they were not. Thus, the mere allocation of attention inward was associated with increased unhappiness. This proposition is also in line with Marchetti et al.’s (2012) findings, which indicate that in a sub-section of participants, reports of being off-task [as opposed to focusing on task performance; a process termed “perceptual decoupling” by Smallwood and Schooler (2006)] were associated with subsequent accessibility of negative thoughts. Negative affect has in fact reliably been associated with the allocation of attention inward also outside the context of rest-related cognition, which further supports the plausibility of these initial propositions. Indeed, Mor and Winquist (2002) describe meta analysis results demonstrating that when attention is focused on the self, negative affect arises. They additionally portray a rich variety of variables, such as gender, psychiatric condition, and type of self-focus (ruminative vs. non-ruminative; private vs. public), which further mediate this association. Importantly, and in accordance with the “perceptual decoupling” hypothesis, self-focused attention is regarded by the authors as the allocation of attentional resources inward, toward internally generated information, as opposed to outward. In yet another context, mental states that explicitly involve the control of attention allocation, such as mindfulness meditation, have been shown to modulate negative emotions (e.g., Taylor et al., 2011; Maron-Katz et al., 2013). Altogether, it could thus be suggested that RRNA depends directly on the allocation of attention resources inward, rather than being an epi-phenomenon of MW. Based on this notion, it could further be suggested that if indeed RRNA arises following the direction of attention from externally driven to internally driven processes, then it would also be expected to depend on whether attentional resources are available for inward allocation.

Based on what is known about rest-related neural mechanisms, several functional neural networks may be suggested to be of relevance to RRNA. The default-mode network (DMN), as well as the executive network (EXE), have both been reliably associated with resting state phenomenology (Andrews-Hanna et al., 2010; Northoff et al., 2010) and with MW in particular (Mason et al., 2007; Buckner et al., 2008; Christoff et al., 2009; Smallwood et al., 2012). Another immediate suspect in the search for neural mechanisms involved in RRNA is the saliency network (SAL; Seeley et al., 2007). Not only has this network been implicated in switching between the executive and default networks as a function of attentional focus (Sridharan et al., 2008), it is also comprised of regions associated with emotion regulation, such as the insula and the anterior cingulate cortex (ACC; Van Dillen et al., 2009; Etkin et al., 2011). Notably, the ACC has been suggested to be functionally segmented (Bush et al., 2000) and parts of it are included in the DMN and EXE as well (Seeley et al., 2007; Laird et al., 2009). In spite of the conceivable relevance of these neural networks to the appearance of RRNA, to our knowledge the relation of these networks, and the neural structures comprising them, to RRNA has not been directly studied.

The current study was designed to examine the conditions under which, during rest, RRNA occurs. Our hypothesis was that availability of attentional resources is a-priori condition for RRNA, and that low attentional resources during rest will be found to result in less negative affect. On the neural level, an exploratory approach was adopted in relation to regions of interest (ROIs) extracted from the default-mode, executive, and saliency networks. To pursue this goal, two simultaneous EEG-fMRI rest scans, a baseline 5-minute scan and a 15-minute rest scan, were collected from a group of healthy volunteers (*N* = 29). Behavioral indicators of affect were additionally collected, at baseline and following the scan session, as well as retrospective reports of MW levels during rest. EEG data was used to calculate the vigilance level of each participant, presumed to influence attentional availability (Dinges et al., 1997; Van Dongen et al., 2003; Mazza et al., 2005). On the basis of parametric EEG assessment, participants were retroactively divided for analysis purposes into two groups of high (i.e., “rest”) and low (i.e., “drowsiness”) levels of vigilance. The change from baseline in fMRI BOLD functional connectivity (FC) between all major hubs of the three examined networks (DMN, EXE, SAL) was also calculated, and then evaluated in terms of their differentiation between vigilance groups and their correlation with behavioral measures.

## MATERIALS AND METHODS

### PARTICIPANTS AND EXPERIMENTAL PROTOCOL

Twenty-nine participants (age: 33 ± 11; 12 females) took part in the study. All participants were healthy, with no neurologic or psychiatric history. Participants provided written informed consent approved by The Tel-Aviv Sourasky Medical Center ethical review board. fMRI data of one participant was discarded from analysis due to extensive head movements; the final fMRI analysis thus included 28 participants.

Prior to scanning, participants first filled out several behavioral questionnaires (specified below). Following, the EEG cap was fitted on their heads and they entered the MRI scanner. The fMRI scan included a 5-minute baseline rest session, a 4-minute masking auditory task and a 15-minute rest session in which high or low vigilance states were examined. The 15-minute rest scan, emphasized to participants as “the main part of the experiment,” was intended to allow a state of reduced vigilance, while the auditory task was used to create an even cognitive load among participants before entering this scan. All scans were performed with eyes closed and both rest scans (5 and 15-minute long scans) included instructions to “lie down and not do anything in particular.”

**FIGURE 1:**
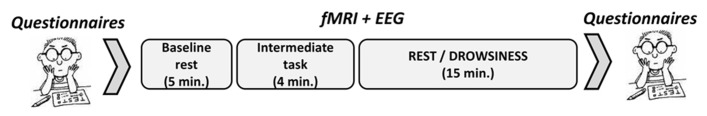
**Illustration of experimental procedure.** Timeline of experimental procedure is illustrated – baseline measurements of behavioral tests (except for MW measurement) were collected from all participants before the fMRI-EEG scan. The fMRI-EEG functional scans included a baseline, 5-minute, rest scan, an intermediate task, and then a 15-minute rest scan. Psychological questionnaires as well as MW report, relating to the 15-minute rest scan, were re-collected upon exiting the MRI scanner.

To allow close proximity of the second behavioral report to participants’ experience during the long rest scan, the later was always recorded last, right before participants exited the scanner. After exiting the scanner, participants were asked on their perceived level of vigilance and filled out questionnaires with the instruction to relate to the main part of the experiment. The study procedure is illustrated in **Figure 1**.

### ASSESSMENT OF MOOD AND THOUGHTS

In order to evaluate the level of mental activity during the long rest scan without interrupting participants’ stream of consciousness we used an indirect measure of MW (Gruberger et al., 2011). Participants were asked to mark on a 10 cm visual analog scale how many thoughts they had during the “main part of the experiment” from “no thoughts at all” to “thoughts all the time.” Participants’ marking on the analog scale were then quantified as a score indicating, from 1 to 10, the experienced occurrence of MW.

To capture change in negative affect following rest, the positive and negative affect scale (PANAS; Watson et al., 1988) was administered at two time points (Levkovitz et al., 2007; Bloch et al., 2010; Marchetti et al., 2012): before the EEG-fMRI scan, relating to their current feeling, and immediately the scan, relating to the main part of the experiment. A Hebrew translation of the PANAS which has been used in previous studies has been selected for this matter (Levkovitz et al., 2007; Bloch et al., 2010). This 20-item questionnaire, designed to trace modifications in emotional state, is comprised of positive-emotion factors and negative-emotion factors. Participants were asked to rate on a scale from one to five the extent to which they felt the emotion presented in each of 20 items. For the purposes of the current study, only negative affect items were selected for further analysis.

### fMRI DATA ACQUISITION AND PREPROCESSING

Imaging was performed on a 3T general electric (GE) Horizon echo speed scanner with a resonant gradient echo planar imaging system (GE, Milwaukee, WI, USA). All images were acquired using a standard head coil. The scanning session included functional T2*-weighted images (FOV = 200 mm, matrix size-64 × 64, voxel size-3 × 3 × 4, TR/TE = 2000/35, Slice thickness = 4 mm, 35 slices without gap, oriented according to the fourth ventricle, flip angle 90°) and a three-dimensional (3D) anatomical scan using T1 SPGR sequence (1 mm × 1 mm × 1 mm).

SPM5 software () was used for image preprocessing as well as voxel-based statistical analysis. The first 20 s of data were discarded to allow steady state magnetization. Functional images were realigned to the first scan and normalized according to standard MNI space. Spatial smoothing was performed utilizing a Gaussian kernel (FWHM = 6 mm).

### EEG DATA ACQUISITION AND PREPROCESSING

EEG data was recorded simultaneously with fMRI acquisition using the MRI compatible BrainAmp-MR EEG amplifier (Brain Products, Munich, Germany) and the electrode cap of BrainCap with sintered Ag/AgCl ring electrodes providing 32 channels: 30 EEG channels, 1 ECG, and 1 EOG channel (Falk Minow Services, Herrsching-Breitbrunn, Germany). The reference electrode was between Fz and Cz. Raw EEG was sampled at 5 kHz. Recording was done using the Brain Vision Recorder software (Brain Products, Munich, Germany).

EEG data underwent the same analysis steps as depicted in our previous work (Ben-Simon et al., 2008, 2012). In brief, two main artifacts were removed: first, artifacts related to the MR gradients were removed from all the EEG datasets using the FASTR algorithm implemented in the FMRIB plug-in for EEGLAB, provided by the University of Oxford Centre for Functional MRI of the Brain, FMRIB (Christov, 2004; Kim et al., 2004). Second, cardioballistic artifacts (QRS peaks) were also removed using the FMRIB plug-in. Following these preprocessing stages, the EEG data were down sampled to 250 Hz and high and low pass filtered at 0.5 and 70 Hz, including a notch filter between 47.5 and 52.5 Hz.

### VIGILANCE CLASSIFICATION ACCORDING TO EEG DATA

An increase in relative alpha (8–12 Hz) power in frontal electrodes is a known marker of decreased vigilance (Gennaro et al., 2005). A further decline in alpha power and a relative increase in lower frequencies (e.g., Theta; 4–7 Hz) mark the next stages of drowsiness which will ultimately lead to sleep onset (Sheldon et al., 2005; Larson-Prior et al., 2009). Based on this rationale, a distinction into vigilance stages based on EEG topographic and spectral information has been utilized (Olbrich et al., 2009). In order to produce an EEG-based electro-physiological estimate of the degree of vigilance among participants, this algorithm was applied on each participant’s EEG data. The classification of vigilance stages was performed in accordance with Olbrich et al. (2009). In brief the following steps were taken:

(1) The pre-processed EEG data was segmented into consecutive 3 s parts and visually inspected for muscular artifacts, fMRI artifact residuals, and sleep spindles which would lead to participant exclusion. Noise contaminated segments were not taken into account in the analysis of the vigilance score and led to the exclusion of one participant due to excessive artifacts (in this case the vigilance classification was set to the “rest” group based on self report). Datasets were further re-referenced to an average electrode calculated as the mean of all 30 channels.
(2) FFT was applied to four main channels (F3, F4, O1, and O2) which are critical in the vigilance assessment algorithm, producing the power in the alpha (8–12 Hz), theta (4–7 Hz), and upper delta (2–4 Hz) rhythms in each second. These power estimations were averaged across 3 s and classified according to the vigilance classification algorithm (Hegerl et al., 2008; Olbrich et al., 2009). In brief this algorithm includes five stages of vigilance based on specific EEG markers: stage 1.1 (i.e., the most alert) was defined as segments in which alpha power at posterior electrodes (O1 + O2) was higher than 55% of the total alpha power at the four examined channels (i.e., F3, F4, O1, O2). If alpha power at frontal channels F3 and F4 was higher than 55% of total alpha power, they were defined as staged 1.3 (lower vigilance stage), while all other possibilities were defined as stage 1.2. Stages 1.4 and 1.5, which denote the lowest vigilance levels before sleep onset, were defined as low frequency power (2–8 Hz) greater than alpha power at the four examined channels.
(3) Mean vigilance scores were computed for each participant in each rest scan. Participants were considered in the “drowsiness” group if their mean vigilance score was higher than the median score (1.14) of all participants during 15-minute rest and their datasets contained at least 5% of reduced vigilance stages (1.2 or higher).

### fMRI ANALYSIS

#### ROI Definition

In accordance with the study hypotheses, major nodes of each of the three functional networks were defined as functional ROIs. For the default mode network these nodes were extracted from a meta analysis by Laird et al. (2009), for the salience network we used a meta analysis by Seeley et al. (2007) and nodes of the executive network were extracted from a meta analysis by Owen et al. (2005). In order to retain high statistical power only the six nodes highest in ALE scores in the meta analyses were chosen for each network, resulting in a total of 18 regions. All nodes were chosen anatomically in accordance with relevant meta-analyses as a 3^3^ voxel box around the main coordinate (see **Table 1**). ROI mean time-course for each participant was estimated by averaging the BOLD time course over all voxels in each of the 18 regions.

**Table 1:** ROIs from the default-mode, executive and salience networks included in the analysis.

Regions	Laterality	MNI coordinates(*x y z*)		
**The default mode network (according to a meta analysis by Laird et al., 2009)**
Precuneus (BA 7,31)	Midline	-4	-62	45
Ventral anterior cingulate	Midline	2	33	-8
Posterior cingulate (BA 31)	Midline	-4	-55	21
Middle temporal gyrus/angular gyrus (BA 39)	R	46	-69	14
Inferior parietal lobule (BA 40)	R	52	-30	25
Middle frontal gyrus (BA 8)	L	-26	14	49
**The executive network (according to a meta analysis by Owen et al., 2005)**
Lateral premotor	R	28	1	54
Dorsal cingulate/medial premotor (SMA)	Midline	-2	10	46
Medial posterior parietal (BA 7)	Midline	10	-70	48
Inferior parietal lobule (BA 40)	L	-36	-53	40
Inferior parietal lobule (BA 40)	R	40	-51	38
Dorsolateral prefrontal (BA 46,9)	R	40	31	34
**The salience network (according to a meta analysis by Seeley et al., 2007)**
Orbitofrontal insula (BA 47,12)	L	-40	18	-12
Orbitofrontal insula (BA 47,12)	R	42	10	-12
Paracingulate (BA 32)	Midline	0	44	28
Dorsal ACC (BA 24)	L	-6	18	30
Hypothalamus	L	-10	-14	-8
Ventrolateral PFC (BA 47)	R	42	46	0

*
Name, laterality, and MNI seed-coordinates of the 18 ROIs which served for analysis. BA, Brodmann area; L, left; R, right; SMA, supplementary motor area; ACC, anterior cingulate cortex; PFC, pre-frontal cortex.*

#### Rest data processing and analysis

In order to examine correlation coefficients in the BOLD signal across the predefined ROIs, we extracted the mean time course for each ROI for both rest scans (baseline and 15-minute scan) for each participant. As common in connectivity analyses (Fox et al., 2005; Tambini et al., 2010), a 128 s high pass filter (0.0078 Hz) was used to remove low frequency trends from each time course using the REST toolbox (Song et al., 2011). Each mean time course was further corrected for the effects of head movements by regression on movement parameters as estimated in the image realignment (movement parameters include three translation and three rotations; Achard et al., 2006). The residuals of these regressions comprised the set of mean time courses used for the correlation analysis. Pearson correlation coefficients were then computed between the BOLD time-course of all ROI pairs in each rest scan for each participant (153 pairs for each participant). In the 15-minute rest condition correlations were computed using a 14-minute time-course due to noise contamination toward the last minute of the scan. All statistics were computed on Fisher *Z*-transformed correlation (*r*) values. *t-*Tests were then conducted on the correlation values of a given ROI pair across rest scans using SPSS software (IBM Corporation, NY, USA). These tests examined which ROI pair’s correlation values showed significant modulation across baseline and 15-minute rest scans. Results were considered significant after controlling for false discovery rate (FDR) at a 5% level (Benjamini and Hochberg, 1995).

## RESULTS

### CHANGE FROM BASELINE IN EEG-BASED VIGILANCE SCORE

**FIGURE 2:**
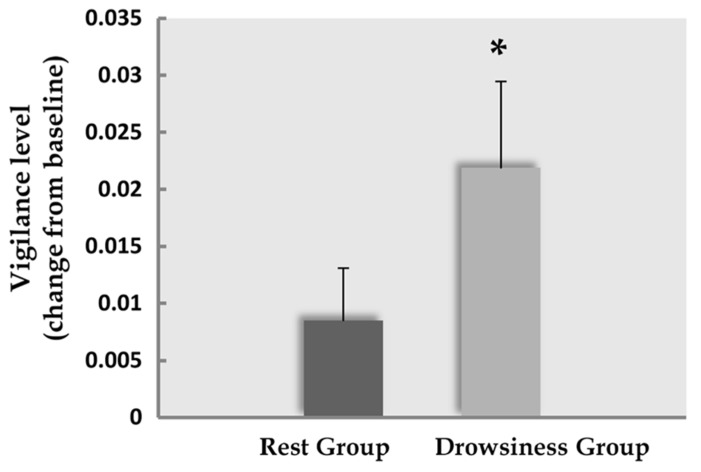
**Decline in EEG-based vigilance scores.** Decline (mean and standard error) in EEG-based vigilance scores (higher score relates to increased drowsiness) between the baseline rest scan and the 15-minute rest scan. Dark column represents the “rest” group; light column represents the “drowsiness” group. A significant decline in vigilance level was found only in the “drowsiness” group. *p < 0.05.

Among participants in the “rest” group, the difference in mean vigilance score between the baseline and the 15-minute rest scans was found to be mildly significant (*M* = 1.12, SD = 0.02 in the 5-minute scan; *M* = 1.13, SD = 0.02 in the 15-minute scan; *t*(14) = -1.86, *p* = 0.084). Within the “drowsiness” group, a significant increase from baseline was found in mean vigilance score of the 15-minute scan (*M* = 1.14, SD = 0.04 in the 5-minute scan; *M* = 1.16, SD = 0.04 in the 15-minute scan; *t*(12) = -2.9, *p* = 0.013; paired-samples *t*-tests; higher score reflecting lower vigilance). These results are depicted in **Figure 2**. Comparing mean vigilance scores across groups revealed mildly significant decreased vigilance already at the baseline scan [*t*(26) = -2.03, *p* = 0.06] in the “drowsiness” group compared to the “rest” group, which decreased further into a significant difference between groups following the 15-minute rest [*t*(26) = -3.0, *p* = 0.008].

### BEHAVIORAL MEASURES

#### Mind wandering

**FIGURE 3:**
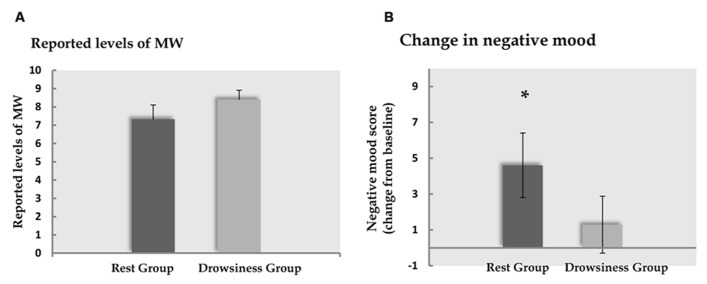
**Behavioral results. Panel A** demonstrates the degree of MW reported by participants to occur during the 15-minute rest scan. **Panel B** demonstrates the change (mean and standard error) from baseline in reports of negative affect following the 15-minute rest scan. Dark column represents the “rest” group; light column represents the “drowsiness” group. Whereas no significant change between groups was found in reported MW, reports within the “rest” group indicated increased negative affect. *p < 0.05.

Five participants (two of the “rest” group, three of the “drowsiness” group) expressed difficulty in quantifying their experienced degree of MW, and were thus instructed to leave this measure empty. Among the remaining participants, reports of participants on the visual analog scale indicated that MW did occur in both “rest” and “drowsiness” groups [*M* = 7.33, SD = 2.81, *t*(12) = 9.41, *p* < 0.001 and *M* = 8.41, SD = 1.66, *t*(10) = 16.83, *p* < 0.001, respectively]. Reports of the degree of MW were not found to be different between groups [*t*(22) = -1.13, *p* = 0.272]. These findings are depicted in **Figure 3A**.

#### PANAS

Cronbach’s alpha coefficients for evaluation of internal consistency within the 10-item negative subscale of the PANAS in the first measurement were found to be at the threshold of marginal reliability (0.63 in the “drowsiness” group and 0.56 in the “rest” group), whereas values following the study manipulation were found to be satisfactory in both groups (“rest” group: 0.87; “drowsiness” group: 0.89). When computed on the change between the first and second measurement, Cronbach’s alpha values were 0.796 and 0.792 for the “rest” and “drowsiness” groups, respectively.

A significant rise from baseline in negative affect as measured by the PANAS questionnaire negative subscale was found among the “rest” group in a paired-samples *t*-test [*M* = 14.60, SD = 2.50 in the baseline measurement; *M* = 19.20, SD = 6.97 in the second measurement; *t*(14) = 2.55, *p* = 0.023], but not in the “drowsiness” group [*M* = 14.79, SD = 3.26 in the baseline measurement; *M* = 16.07, SD = 7.04 in the second measurement; *t*(13) = 0.81, *p* = 0.434]. These findings are depicted in **Figure 3B**. The difference between groups in the degree of change from baseline was found to be mildly significant [*t*(27) = 1.37, *p* = 0.091], with a trend toward the “rest” group reporting a greater rise in negative affect compared to the “drowsiness” group.

Though this study’s main interest was in RRNA, the PANAS positive subscale was analyzed as well in order to check the specificity of the effect of the study manipulation to negative affect. PANAS positive subscale scores were found to change in both groups in a manner which showed no sensitivity to vigilance level. Although a significant decline in positive scores was found in both groups [*M* = 30.87, SD = 4.90 in the baseline measurement; *M* = 24.20, SD = 4.96 in the second measurement; *t*(14) = 4.35, *p* = 0 < 0.001 in the “rest” group; *M* = 30.93, SD = 5.18 in the baseline measurement; *M* = 22.71, SD = 6.19 in the second measurement; *t*(13) = 4.21, *p* = 0 < 0.001 in the “drowsiness” group], no significant difference was found between the groups in the degree of change from baseline [*t*(27) = 0.66, *p* = 0.515].

### fMRI CONNECTIVITY RESULTS

To investigate the neural modulation associated with RRNA we first explored the ROI correlations within the “rest” group. Out of all ROI pairs examined (*N* = 153), a significant change from baseline was found only between the dorsal cingulate cortex/medial premotor (SMA) component of the EXE, and the ventral ACC component of the DMN revealing a threshold-level significant increase in correlation [*M* = -0.08, SD = 0.16 in the 5-minute scan; *M* = 0.17, SD = 0.15 in the 15-minute scan; *t*(13) = 4.84, FDR corrected *q* = 0.05]. In the “drowsiness” group, this correlation did not appear to undergo a significant change [*M* = 0.13, SD = 0.15 in the 5-minute scan; *M* = 0.04, SD = 0.16 in the 15-minute scan; *t*(13) = 2.4, *q* = 0.11; paired-samples *t*-tests].

**FIGURE 4:**
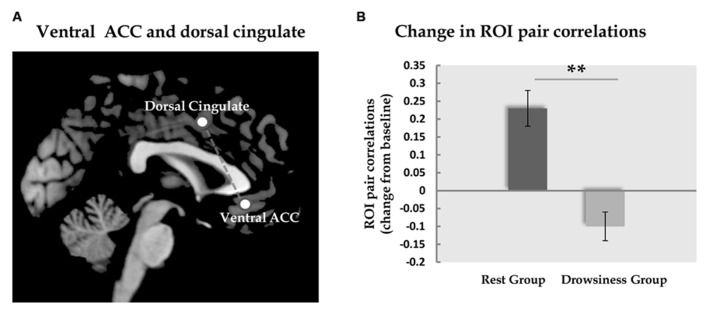
**Functional connectivity between dorsal and ventral ACC. Panel A** demonstrates the location of the dorsal and the ventral ACC ROIs analyzed in the current study. **Panel B** shows the change (mean and standard error) in correlation between these two ROIs from the baseline rest scan to the 15-minute rest scan. In the “rest” group, but not in the “drowsiness” group, a significant rise in correlation was found. Dark column represents the “rest” group; light column represents the “drowsiness” group. **q < 0.01.

Across groups, the same pair of regions, the dorsal cingulate cortex and the ventral ACC, was the only pair in which the difference in correlation in the long rest scan relative to the baseline scan was found to differ significantly between the “rest” and “drowsiness” groups [*M* = 0.25, SD = 0.19 and *M* = -0.10, SD = 0.15, respectively; *t*(26) = 5.30, *q* = 0.002]. None of the other pairs was found to significantly differentiate between groups following correction for multiple comparisons. These findings are depicted in **Figure 4**.

### CORRELATIONS BETWEEN fMRI AND BEHAVIORAL REPORTS

**FIGURE 5:**
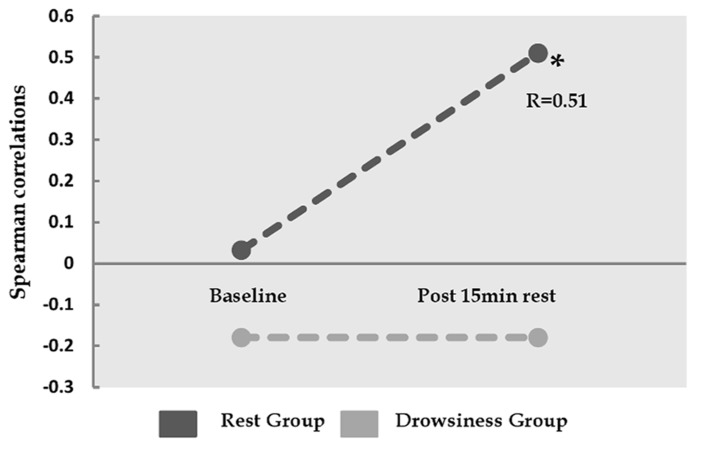
**Relation between the degree of correlation within dorsal and ventral ACC, and reported negative affect.** *Y*-axis represents Spearman correlation. Whereas the correlation between ventral ACC and dorsal cingulate was not found to be associated with negative affect at baseline, these two factors show significant correlation among the “rest” group, but not among the “drowsiness” group, following the 15-minute rest session. *p < 0.05.

Whereas no correlations were found between baseline FC in the dorsal-ventral ACC pair and baseline negative affect measurements, a significant positive correlation (*r* = 0.51, *p* = 0.037) was found between FC within this pair during 15-minutes rest and post scan reports of negative affect in the “rest” group, but not in the “drowsiness” group (*r* = -0.18, *p* = 0.476). This finding is demonstrated in **Figure 5**.

Correspondingly, the increase from baseline FC in dorsal-ventral ACC was found to be positively correlated with the increase in reports of negative affect among participants in the “rest” group in a mildly significant manner (*r* = 0.4, *p* = 0.07). An opposite trend was found among participants in the “drowsiness” group, which tended toward a negative correlation in change from baseline of these two variables (*r* = -0.43, *p* = 0.064).

## DISCUSSION

In the current study we hypothesized that RRNA would be found to occur to the extent that attentional resources are available for inward allocation. Our findings support this hypothesis, revealing that negative affect did not appear to rise when levels of vigilance decreased while replicating the finding of RRNA among participants who remained vigilant during rest. The two groups were further found to differ in degree of modification of FC between specific nodes of the executive and the default-mode networks. Moreover, the degree of this functional connection was found to be correlated with the degree of negative affect only among the vigilant “rest” group. To our knowledge, this is the first demonstration of a dramatic difference in occurrence of rest-related negative affect by simply monitoring the degree of vigilance.

Segregating participants into two groups according to their vigilance levels during a 15-minute rest scan revealed that indeed, and in accordance with the study hypothesis, the rise in negative affect following rest was dependent on a maintained level of vigilance: when vigilance was diminished, negative affect level did not increase. First and foremost, this finding serves as evidence that rest-related negative affect is not a necessary outcome of the awake resting state. Once this important dissociation is empirically established, it is intriguing to examine which factors, in both neural and psychological terms, stem from the current findings as mediating the occurrence of RRNA.

First, vigilance is demonstrated in the current study as a mediating factor of the occurrence of negative affect. Assuming that reduced vigilance in fact resulted in lower availability of attentional resources (Mazza et al., 2005), one could suggest that this availability, which is naturally required for focusing attention inward, is a pre-requisite of RRNA. The question still remains, however, whether MW plays a role in this causal chain; it could be argued that negative affect is not a mere result of internally directed attention, but rather a result of heightened attention toward MW processes [also termed “meta-awareness” (Schooler and Schreiber, 2004; Christoff et al., 2009)]. Importantly, in our study MW was reported to occur in both groups, indicating that participants were not too drowsy to engage in MW. Thus, our findings could serve as a basis for suggesting a mechanism in which, when vigilance is maintained, MW is in fact more attended to and therefore more self-monitored, whereas during lowered vigilance, MW occurs without superior monitoring processes resulting in reduced negative affect. To quantify whether, and to what degree, MW is a mediating factor in the association between allocation of attention inward and RRNA, further studies are required which assess the degree and the contents of MW in greater resolution, for instance by using real-time thought sampling methods (Mason et al., 2007; Christoff et al., 2009; McVay et al., 2009) and MW questionnaires (Delamillieure et al., 2010; Michael et al., 2013). Nevertheless, the current findings provide novel indication of the dependence of RRNA on availability of internally allocated attention, and not on MW alone.

Additional insights into the mechanisms underlying RRNA stem from the current findings of neural differences between high and low vigilance resting states. To begin with, a notable finding is that both rest conditions (high and low levels of vigilance) were found to share many neural commonalities: out of the three networks inspected, including a total of 153 ROI pairs, only one specific functional connection was found to be significantly different across low and high vigilance conditions. This pair of regions includes the subgenual, ventral ACC, extracted from the DMN (Laird et al., 2009), and the posterior part of the supragenual, dorsal ACC, an element of the executive network (Owen et al., 2005). FC between these two sub-regions of the ACC was found to undergo a dramatic amplification during the rather long rest session, only in the high vigilance condition. This finding was not detected in any of the other examined neural connections, and thus cannot be explained merely by the global attenuation of neural resources or by general changes in neural connectivity related to drowsiness (Van Someren, 2011). It is also worth noting that the amplification of FC between the two sub-regions of the ACC was found in a 15-minute rest session, in comparison to a 5-minute rest session. It thus appears that some rest-related neural processes do not take place immediately, but rather require longer periods of rest to occur. This is an important finding that indicates a qualitative difference in neural activity patterns, and possibly in the corresponding phenomenology, between long and short rest sessions. Though this matter has received relatively little attention in the context of rest related phenomenology, it has been suggested in the wider context of consciousness related phenomena that the spatio-temporal evolvement of neural processes, as well as the degree of their linearity over time, is of crucial significance (Lundervold, 2010). Different types of rest-related phenomenology might thus further be dependent on the duration of rest for their evolvement, though this notion too requires further systematic scientific inquiry.

Functionally, the ventral, subgenual ACC has been described as a critical node in self and emotional related mental processing (Gusnard et al., 2001; Kircher et al., 2002; D’Argembeau et al., 2005; Etkin et al., 2011), and the posterior, supragenual ACC has been shown to be involved in action monitoring of both the self and others (Mueller et al., 2007; Demanet et al., 2013). Indeed, both regions have been reported as related to cognitive appraisal of emotional stimuli in a dual, inter-related process in which cognitive processing mainly takes place in the dorsal regions of the MPFC while the ventral regions mediate the affective domain (Bush et al., 2000), though this dichotomy has been recently challenged (Etkin et al., 2011). Similarly, this model echoes known findings, in which the dorsal ACC has been suggested to represent a hierarchically superior process of active, engaged, monitoring (Duncan and Owen, 2000; Lemogne et al., 2011). Thus, the current finding that the alert, but not the drowsy, resting state is characterized by increased FC between these two regions provides first of a kind neural reflection of the proposition that the role of internally allocated attention at rest in the emergence of negative affect might be related to monitoring of internal mental activity. Importantly, this notion is reinforced by the finding that the sharp rise in FC between these two nodes was further correlated with the rise in negative affect only in the vigilant “rest” group.

The finding that RRNA is mediated by attentional processes and, when increased, is associated with increased connectivity in dorsal and ventral ACC heightens the potential relevance of the degree of engagement of the self in rest related mental activity to the outcome experience. Indeed, both dorsal and ventral ACC have been implied as major neural hubs of self-referential mental activity, and even more so in the processing of self-referential information which also carry additional emotional aspects (Northoff and Bermpohl, 2004; Northoff et al., 2006; Demanet et al., 2013). Self-focused attention, which notably is a different theoretical construct from meta-awareness (Schooler and Schreiber, 2004; Christoff et al., 2009), has been demonstrated by numerous studies to result in negative feeling (Mor and Winquist, 2002). In correspondence, studies of the beneficial affective outcome of meditation demonstrate a reduction in neural recruitment of dorsal ACC (Gard et al., 2012; Maron-Katz et al., 2013). The theoretical proposition that the association demonstrated in the current study between high vigilance and negative affect at rest might be mediated by heightened attention to MW is thus further supported by the finding of the involvement of self related neural regions in the process, as MW is primarily a self-oriented mental function (Gusnard, 2005; Baars, 2010). Why would negative affect arise following self-focused cognition? A potential explanation which has been suggested relates to the evolutionary pressure to plan and think ahead (Buckner et al., 2008; Mooneyham and Schooler, 2013). According to this proposition, MW is oriented toward the process of acute self-related problem solving; orientation of attention toward this process would naturally result in stronger distress. Techniques that practice lowering this self-engagement are thus expected to be emotionally beneficial. Indeed, while some have suggested that meditation, in this case mindfulness meditation, is an opposing structure to MW altogether (Mrazek et al., 2012), many other eastern traditions attribute the benefits of meditation practice to a diminished involvement in the habitual mode of self-reference (Cahn and Polich, 2006; Ott et al., 2010). Similarly, in the current study reduced vigilance might have involved less involvement of the self in rest-related mental activity, which, to a certain extent like meditation states, resulted in less dorsal-ventral ACC neural engagement, and in turn less negative affect.

The psychological and neural differences between high and low vigilance groups in the current study thus point toward two potentially parallel scenarios of rest related neural and mental states. While MW may occur in both scenarios, the alert resting state might be characterized by stronger activity in attentional-emotion self-monitoring neural circuits, resulting in heightened negative affect. In contrast, a more drowsy resting state might not involve this aspect of activation, resulting in a less negative emotional experience. This proposition is in fact in line with the finding that when participants are found to be off-task during task performance, in or outside the laboratory, negative affect is reported (Michael et al., 2013; Mooneyham and Schooler, 2013); possibly due to their being off task while relatively active and alert.

Several issues need to be addressed as limitations of the current study. First, we used a subjective measure of MW administered retroactively at the end of the fMRI-EEG scan. The current study was designed to collect relatively long uninterrupted fMRI and EEG scans, based on the assumption that longer rest sessions are required to create identifiably different patterns of rest, which can later be compared to each other in terms of vigilance, neural connectivity, and psychological phenomenology. In order to enable the occurrence of undisturbed rest, all reports in this study, including that of mind wandering, had to be obtained retroactively. Nevertheless, such reports may have compromised the accuracy of MW levels in comparison with real-time thought sampling or other task related measurements. Secondly, we used an objective, yet indirect measure of attention allocation by examining known EEG parameters of vigilance, given that drowsiness leads to a lower availability of attentional resources. Future studies could use a combined approach of rest and parametric attention manipulations (for instance using a task with several degrees of cognitive load) to directly examine the role of attention allocation in shaping RRNA. Finally, the current study focused on the affective experience induced by a long session of rest while taking vigilance state into account. For a more comprehensive explanatory model, empirical examinations of other possible factors (e.g., physiological arousal, relevant personality traits, the content of MW, etc.) could further illuminate the nature of the interplay between attention allocation and RRNA.

To conclude, our findings support, both on the behavioral and on the neural level, the proposition that rest-related negative affect is associated with allocation of attention inward. This finding echoes known models of emotion regulation and reappraisal emphasizing the crucial role that attention and meta-awareness play in affective processing. Similar with the meditation practice of letting thoughts go, it seems that the less we monitor our internal world, the happier we are.

## Conflict of Interest Statement

The authors declare that the research was conducted in the absence of any commercial or financial relationships that could be construed as a potential conflict of interest.
